# Is There Geographic Variation in Poland in the Distribution of Specific High-Risk HPV Genotypes in Normal Cervical Epithelium, Dysplastic Lesions, and Cervical Cancers?

**DOI:** 10.3390/cancers18111774

**Published:** 2026-05-28

**Authors:** Beata Biesaga, Anna Mucha-Małecka, Anna Kruczak, Wiktor Szatkowski

**Affiliations:** 1 Department of Tumor Pathology, Maria Skłodowska-Curie National Research Institute of Oncology, Kraków Branch, 31-115 Kraków, Poland; anna.kruczak@krakow.nio.gov.pl; 2 Department of Radiotherapy, Maria Skłodowska-Curie National Research Institute of Oncology, Kraków Branch, 31-115 Kraków, Poland; anna.mucha@krakow.nio.gov.pl; 3 Department of Gynecologic Oncology, Maria Skłodowska-Curie National Research Institute of Oncology, Kraków Branch, 31-115 Kraków, Poland; wiktor.szatkowski@krakow.nio.gov.pl

**Keywords:** HPV genotypes, geographical variation, Poland

## Abstract

Cervical cancer remains a major public health challenge worldwide and in Poland, where mortality rates are among the highest in the European Union. Persistent infection with high-risk human papillomavirus (hrHPV), particularly genotype HPV16, plays a central role in cervical carcinogenesis. This study aimed to evaluate the regional variability of hrHPV prevalence and genotype distribution in Poland and to assess its potential implications for cervical cancer incidence and prevention strategies. A systematic literature review was conducted using PubMed, Scopus, and Google Scholar to identify studies published up to May 2025 reporting hrHPV prevalence and genotypes among Polish women. Eligible studies included population-based cohorts, women undergoing screening, and patients with cervical lesions or cancer. The analysis revealed substantial heterogeneity in hrHPV prevalence and genotype distribution across regions and study populations. Nationwide data indicate high overall HPV prevalence (up to 50.9%), with HPV16 consistently dominating, followed by HPV31, HPV51, HPV52, and HPV66. Regional differences were observed, including a higher prevalence of HPV51 in southern Poland and HPV56 and HPV45 in central regions. Studies in women with abnormal cytology or cervical cancer showed markedly higher hrHPV prevalence (often >90%), with HPV16 predominating in high-grade lesions and invasive cancer. These findings confirm the dominant oncogenic role of HPV16 while highlighting significant regional variability in other hrHPV genotypes. Such differences may influence the effectiveness of screening and vaccination programs. Strengthening standardized, regionally stratified HPV surveillance is essential to optimize cervical cancer prevention and tailor public health interventions in Poland.

## Simple Summary

Cervical cancer is a significant health problem in Poland, largely caused by infection with high-risk types of human papillomavirus (hrHPV). This study summarizes current knowledge about how often different HPV types occur in Polish women and whether their distribution varies between regions. A review of published studies shows that HPV16 is the most common and most dangerous type across all groups of women, including those with normal cytology, precancerous lesions, and cervical cancer. However, other hrHPV types, such as HPV31, HPV51, HPV52, HPV56, and HPV66, also occur frequently, and their distribution differs by region. For example, HPV51 is more common in southern Poland, while HPV56 and HPV45 are more often found in central regions. These regional differences may affect the effectiveness of screening and vaccination programs. Better, standardized monitoring of HPV types across Poland is needed to improve prevention and early detection of cervical cancer.

## 1. Introduction

Cervical cancer is nowadays the fourth most common cancer among women worldwide. In 2022, approximately 660,000 new cases were diagnosed globally, with around 350,000 deaths resulting from this disease [1]. According to data from the Polish National Cancer Registry in 2022, there were approximately 2200 new cases of cervical cancer diagnosed, and nearly 1500 deaths attributed to this type of cancer [2]. The standardized incidence rate is estimated at around 10 per 100,000 women, while the standardized mortality rate is approximately 5.5 per 100,000. These data place Poland among the countries with one of the highest cervical cancer mortality rates in the European Union. The discrepancy between incidence and mortality highlights a delay in diagnosis due to, on one side, limited access to early detection methods and, on the other side, low attendance of Polish women at the screening program.

In the pathogenesis of cervical dysplasia and carcinoma, Human papillomavirus (HPV) infection plays a central role, particularly with high-risk genotypes. HPVs are a large group of DNA viruses, with over 200 identified genotypes, of which more than 40 can infect the anogenital tract. The International Agency for Research on Cancer (IARC) has classified HPV genotypes into categories based on their oncogenic potential, primarily with respect to their association with cervical and other anogenital cancers [3,4]. According to this classification, HPV genotypes are divided into the following risk categories: (1) group 1: carcinogenic to humans (high-risk HPV—hrHPV), including: HPV16, 18, 31, 33, 35, 39, 45, 51, 52, 56, 58, and 59; (2) group 2A: probably carcinogenic to humans with limited evidence of carcinogenicity, but strong mechanistic and epidemiological support: HPV68; (3) group 2B: possibly carcinogenic to humans with limited and less consistent evidence: HPV26, 53, 66, 67, 70, 73, and 82; (4) group 3: not classifiable as to their carcinogenicity in humans and (5) low-risk HPV (lrHPV) types with genotypes not associated with cancer but are known to cause benign lesions [5].

HPV infection among women with normal cervical cytology constitutes a significant public health burden. Osmani et al. (2025), analyzing 73 studies with 41,745 women aged ≥50, reported a global prevalence of any HPV of 11.7% and high-risk HPV of 6.45% [6]. Earlier analyses found similar levels: Bruni et al. (2010) reviewed 194 studies including 1,016,719 women, reporting 11.7% [7], and de Sanjosé et al. (2007) found 10.4% among 157,879 women [8]. It also seems that hrHPV prevalence varies regionally, with the highest in Sub-Saharan/Western Africa (16.5–32.2%), Central/South America (10.4–24.5%), and Eastern Europe (21.4–29.1%) [6,7,8,9], intermediate in Asia (3.3–14%), and lowest in Western Europe (3.7–6.6%) [6,7,9]. Globally, high-risk HPV16 dominates (1.2–19.7%), followed by HPV18, 31, 52, and 58, with other HR-HPVs showing regional patterns: HPV53/51 in North America, HPV58 in Asia/Latin America, HPV31/33 in Europe, and high genotype diversity in Africa [6,7,8,9]. In women with LSIL, HPV16 remains most prevalent (26.3%), followed by HPV31, 51, 53, and 56, with regional variation: HPV16 16.3–32.6%, HPV18 5.0–11.5%, and certain genotypes are more frequent in specific regions (HPV31/33 in Europe, HPV58 in Asia) [10]. However, these results should be interpreted with caution, particularly in light of substantial differences in age distributions across study populations, as some analyses included only selected age groups, limiting direct comparability. In addition, variations in screening strategies and HPV detection methodologies further reduce the reliability of direct temporal comparisons. Importantly, in the context of ongoing HPV vaccination programs, prevalence estimates may change over time, and observed rates should therefore not be considered stable but rather dynamic, potentially evolving in response to vaccination coverage and population-level immunity. The apparent similarity between studies is likely driven by substantial heterogeneity in study populations, including differences in age structure, screening context, and HPV detection methods, which limits direct comparability. In particular, the restriction to women aged ≥50 years in Osmani et al. makes direct comparison with earlier analyses based on broader age ranges problematic [6]. In addition, temporal differences in HPV vaccination coverage and the implementation of organized screening programs may influence observed prevalence patterns, particularly in younger cohorts, further complicating cross-study comparisons. Post-vaccination meta-analyses have demonstrated substantial changes in HPV epidemiology in countries with high vaccine coverage. A large systematic review and meta-analysis by Drolet et al. [11], including data from multiple world regions, showed marked reductions in vaccine-targeted HPV types (HPV16/18), with decreases of up to 80% in adolescent girls within 5–8 years following vaccine introduction. The study also demonstrated significant herd effects, with reductions observed among unvaccinated populations, indicating a decline in overall HPV transmission at the population level. Consistent findings have been reported in long-term population-based studies, such as Falcaro et al. [12], which showed substantial reductions in both HPV-related precancerous lesions and cervical cancer incidence following the implementation of HPV vaccination. In vaccinated cohorts, cervical cancer incidence was reduced by nearly 90%, underscoring the strong preventive impact of vaccination on clinically relevant outcomes. Additional pooled analyses and systematic reviews conducted in the post-vaccination era consistently report significant declines in HPV16/18 prevalence in countries with established immunization programs, along with reductions in related clinical endpoints such as anogenital warts [13]. Overall, these findings indicate that HPV epidemiology is dynamic rather than stable and is strongly influenced by vaccination coverage, population immunity, screening practices, and diagnostic methodologies. Therefore, comparisons of HPV prevalence across different time periods and study populations should be interpreted with caution, as apparent similarities may reflect methodological and demographic differences rather than true epidemiological stability.

In CIN (Cervical Intraepithelial Neoplasia) 2/3 and invasive cervical cancer (ICC), HPV16 and HPV18 predominate, with combined attributable fractions of 71.9–83.2% across regions [14]. HPV16 prevalence in ICC ranges 52–79%, with regional contributions from other genotypes: HPV52/58 in East/Southeast Asia, HPV33/66 in Central/South America, HPV35 in Africa, and HPV45 is frequently detected in adenocarcinomas [15,16,17,18,19,20]. In Europe, HPV16/18 remains the most common oncogenic type in ICC and high-grade squamous intraepithelial lesion (HSIL). However, their distribution varies by subregion due to behavioral, genetic, and public health factors [21]. Combined HPV16/18 prevalence in ICC is 74–77%, with hrHPVs 31, 33, and 45 showing regional variation. Eastern and Southeastern Europe exhibit higher HPV16/18 prevalence and broader HR-HPV diversity. Multicenter studies of cervical glandular neoplasia report HPV16 prevalence 15.8–75%, HPV18 25–68.4%, and HPV45 0–16.7% [22], while Tjalma et al. found HPV16 in 63% of ICC cases, HPV18 15.2%, and HPV45 5.3%, illustrating type-specific progression risks relevant for screening strategies [23].

Considering Europe, to the best of our knowledge, there are currently no direct, systematically designed studies providing conclusive evidence of geographic differences in hrHPV genotype distribution among women in particular countries. The available data are mostly indirect and derived from heterogeneous studies with different populations and methodologies. However, observations from other European countries with similar demographic structures suggest that, in countries such as Germany [24], Italy [25,26], and Spain [27], geographical variation in the distribution of hrHPV genotypes may occur. These variations may be attributed to multiple interrelated factors, including disparities in access to healthcare, differences in sexual behaviors and the average age of sexual debut, unequal coverage of HPV vaccination programs, and variable uptake of both cytological and molecular screening strategies. Poland exhibits substantial regional heterogeneity that could influence the distribution of hrHPV types. Differences in urbanization levels, economic development, the organization and accessibility of gynecological care, and health literacy across regions in Poland may also contribute to local variations in HPV epidemiology. Importantly, these disparities are also reflected in the incidence of cervical cancer. Epidemiological data show that the burden of cervical cancer in Poland is not uniformly distributed; rather, incidence rates vary significantly between voivodeships. The highest incidence rates have been reported in voivodeships such as Świętokrzyskie, Lubelskie, and Podkarpackie, where the burden of disease remains significantly elevated compared to the national average [2]. In contrast, voivodeships including Mazowieckie, Wielkopolskie, and Pomorskie consistently demonstrate some of the lowest incidence rates of cervical cancer. These variations suggest differences in regional risk factors, access to preventive services, screening coverage, and possibly socio-economic determinants that influence disease occurrence.

Recommendations of the Polish Society of Gynecologists and Obstetricians (PTGiP), together with national expert consensus statements, support HPV-based prevention strategies. For example, joint expert guidelines from PTGiP and the Polish Society of Colposcopy and Cervical Pathophysiology emphasize modern diagnostic approaches that integrate HPV testing into cervical cancer prevention and management algorithms [28]. These recommendations are consistent with the current organization of the national screening program in Poland, in which high-risk HPV testing has been introduced as a primary or first-line screening modality, with cytology used as a triage method. In light of these recommendations, understanding the epidemiology of specific HPV genotypes within the local population has become crucial. In Poland, there is a lack of systematic analysis of whether significant geographic variation in HPV genotype distribution exists. Such differences may affect the sensitivity and effectiveness of HPV-based screening tests, the design of region-specific public health strategies, the risk assessment for progression to HSIL/CIN2, and the justification for tailoring diagnostic tools to regional needs (e.g., locally adapted test panels). Therefore, the primary aim of this study is to provide a comprehensive, systematically synthesized overview of the regional distribution of high-risk human papillomavirus genotypes among Polish women, based on all available published data up to May 2025. Unlike previous reports focusing on individual cohorts or isolated regions, this review integrates evidence from diverse populations across Poland into a unified, nationally stratified framework, enabling direct comparison of hrHPV genotype patterns between regions and clinical subgroups.

A key novel aspect of this work is the consolidation of fragmented regional data that have not previously been analyzed collectively, allowing the identification of consistent but previously under-recognized geographic differences in non-HPV16 genotypes. In addition, the study aims to assess whether these regional variations may have implications for the performance and optimization of cervical cancer screening strategies and HPV vaccination programs in Poland.

Ultimately, this study fills an important gap in the literature by delivering the first nationwide, regionally structured synthesis of hrHPV epidemiology in Poland, providing clinically relevant insights that may support more targeted and evidence-based public health interventions.

## 2. Literature Search

A literature search was conducted to identify studies reporting the prevalence and genotype distribution of high-risk human papillomavirus (hrHPV) among women in Poland. The search aimed to include both population-based screening studies and studies focused on women with cytological abnormalities or cervical cancer.

**Figure 1:**
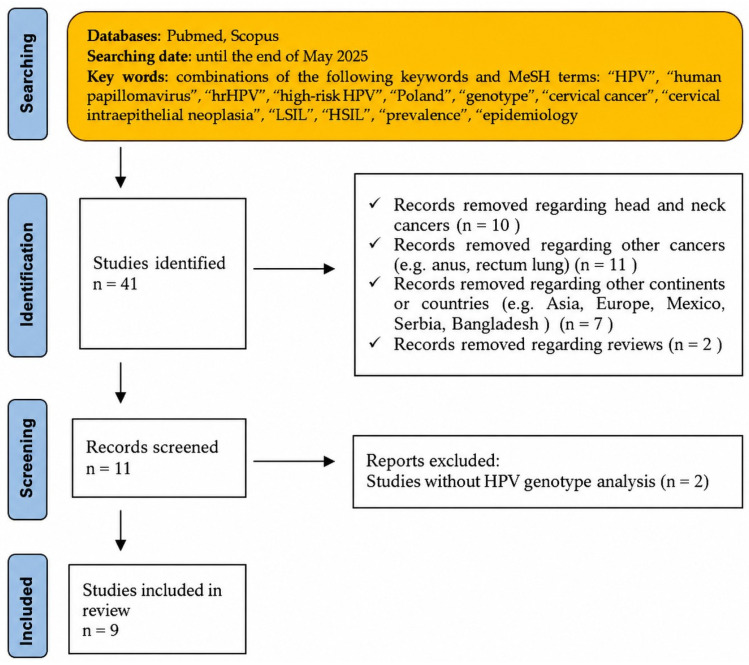
Flow diagram for structured study selection process. Abbreviations: HPV, Human Papillomavirus; hrHPV, High-Risk Human Papillomavirus; HSIL, High-Grade Squamous Intraepithelial Lesion; LSIL, Low-Grade Squamous Intraepithelial Lesion; MeSH, Medical Subject Headings.

The literature review was performed using the following electronic databases: PubMed and Scopus (Figure 1). The search included publications available up to May 2025 and used combinations of the following keywords and MeSH terms: “HPV”, “human papillomavirus”, “hrHPV”, “high-risk HPV”, “Poland”, “genotype”, “cervical cancer”, “cervical intraepithelial neoplasia”, “LSIL (Low-Grade Squamous Intraepithelial Lesion)”, “HSIL”, “prevalence”, and “epidemiology”. Boolean operators such as AND, OR, and NOT were applied to refine the search.

Inclusion criteria for the selection of articles were as follows:

1. Original research articles published in peer-reviewed journals
2. Studies conducted in Poland
3. Studies reporting quantitative data on hrHPV prevalence and/or genotype distribution
4. Studies including general female populations, women undergoing gynecological screening, or women diagnosed with cervical lesions or cancer
5. Articles published in English or Polish

Exclusion criteria were as follows:

- Review articles, conference abstracts, case reports, and editorials
- Studies with insufficient data on HPV genotype distribution
- Studies using outdated or non-specific HPV detection methods without genotype resolution

## 3. Results

### 3.1. High Risk Human Papillomavirus Prevalence and Genotype Distribution in the General Female Population of Poland

A large nationwide retrospective study by Glinska et al. [29] analyzed over 30,000 HPV test results obtained in Poland prior to the implementation of the population-based HPV vaccination program, providing one of the most comprehensive baseline datasets on HPV genotype distribution in the country. Among 11,151 women with complete genotyping, 50.9% were HPV-positive, and high-risk HPV (hrHPV) infection was detected in 39.0% of cases. HPV16 was the most frequently identified genotype (24.0% of all HPV-positive women), followed by HPV31 (11.3%), HPV66 (11.3%), HPV53 (10.5%), and HPV51 (10.0%). When restricting the analysis to hrHPV-positive women, HPV16 remained dominant (31.3%), with HPV31 and HPV66 showing similarly high prevalence (14.7% each), followed by HPV51 (13.0%) and HPV56 (11.0%). The study further demonstrated age-related variability in HPV distribution, with higher overall HPV positivity in younger women and a progressive decline in total infection rates with increasing age. Meanwhile, hrHPV infections showed a more complex, genotype-dependent pattern that was less strictly age-dependent, although HPV16 remained consistently the dominant type across all age groups. Despite a large, geographically broad sampling frame that included patients recruited from all 16 voivodships of Poland, the study did not perform a stratified analysis of hrHPV prevalence by geographic region. As a result, potential regional differences in HPV genotype distribution within Poland could not be assessed. Due to its methodological design, which relies on aggregated laboratory data, this study does not provide stratified clinical information linking HPV status to cytological categories (normal vs. abnormal cytology). Therefore, it does not allow for a direct comparison with studies included in Table 1, Table 2 and Table 3 of our manuscript, which require clearly defined clinical subgroups.

Overall, the findings confirm established epidemiological patterns, including higher overall HPV prevalence in younger women, likely reflecting increased exposure following sexual debut, and a gradual decline in total infections with age, consistent with spontaneous viral clearance. In contrast, hrHPV infections tend to persist longer and show a more heterogeneous age-related distribution, reflecting their clinical relevance and stronger association with persistent infection rather than transient exposure. Due to its methodological design, which relies on aggregated laboratory data, this study does not provide stratified clinical information linking HPV status to cytological categories (normal vs. abnormal cytology). Therefore, it does not allow for a direct comparison with studies included in Table 1, Table 2 and Table 3 of our manuscript, which require clearly defined clinical subgroups.

### 3.2. High Risk Human Papillomavirus Prevalence and Genotype Distribution in the Female Population Without Cervical Abnormalities Across Different Regions of Poland

Table 1 summarizes data derived from three Polish studies evaluating the prevalence of hrHPV infection and genotype distribution among women without histopathological cervical lesions or cytological abnormalities. The analyzed studies, conducted between 2008 and 2023, encompass diverse geographic regions of Poland and varying diagnostic methodologies, thereby providing a broad and representative overview of hrHPV epidemiology in the Polish female population. In the cohort from the Wielkopolska Region (central–western Poland), comprising women without histopathological abnormalities (*n* = 246), hrHPV infection was relatively infrequent and characterized by a heterogeneous distribution of genotypes [30]. HPV16 was the most frequently detected genotype (10.2%), followed by HPV53 and HPV66 (each 5.7%), HPV31 (4.9%), HPV52 (4.1%), and HPV51 (1.2%). Notably, no single genotype demonstrated clear epidemiological dominance, suggesting a dispersed pattern of hrHPV circulation in this population. In contrast, earlier studies conducted in 2008 reported a generally lower prevalence of hrHPV infection. In a population of women without cervical abnormalities from the Warsaw region, Bardin et al. identified hrHPV infection in 9.9% of participants, with HPV16, HPV56, HPV45, HPV31, and HPV52 being the most frequently observed genotypes [31]. Similarly, Szostek et al., analyzing women with normal cytology from south-central Poland, reported a higher overall hrHPV prevalence of 21.0% [32]. In this cohort, the most common genotypes were HPV51, HPV52, HPV45, HPV56, and HPV66.

**Table 1:** Regional Distribution and Prevalence of High-Risk HPV Genotypes in the Female Population Without Cervical Abnormalities across Poland.

Authors (Year of Publication)	Cohort	Geographical Region	Material	Method of HPV Detection	hrHPV Prevalence
Przybylski et al. (2023) [30]	246 women without cervical abnormalities tested between 2018 and 2022	Wielkopolska Region (central–western Poland)	Cervical material	HPV DNA Immunoassay with oligoprobe cocktails, detection of 37 HPV genotypes	**General percentage of hrHPV positivity:** **no data** HPV16—10.2% HPV53—5.7% HPV66—5.7% HPV31—4.9% HPV52—4.1% HPV51—1.2%
Bardin et al. (2008) [31]	779 women without cervical abnormalities tested in 2006	Warsaw region	Exfoliated cervical cells	HPV DNA Immunoassay with oligoprobe cocktails, detection of 44 HPV genotypes	**General percentage of hrHPV positivity:** **77 (9.9%)** HPV16—22.5% HPV56—10.1% HPV45—9.4% HPV31—8.7% HPV52—8.7% HPV33—6.5% HPV58—5.1% HPV18—4.3% HPV73—4.3% HPV35—2.2% HPV39—2.2% HPV59—2.2% HPV68—2.2% HPV82—2.2%
Szostek et al. (2008) [32]	42 women without cervical abnormalities tested between: no data	Krakow region	Cervical cytology material	INNO-LiPA HPV Genotyping	**General percentage of hrHPV positivity:** **9 (21.0%)** HPV51—55.6% * HPV52—22.2% HPV45—11.1% HPV56—11.1% HPV66—11.1%

Abbreviations: HPV, Human Papillomavirus; hrHPV, High-Risk Human Papillomavirus. * The sum of percentages exceeds 100%, which reflects the presence of multiple HPV infections per individual.

### 3.3. High Risk Human Papillomavirus Prevalence and Genotype Distribution in the Female Population with Cervical Abnormalities Across Different Regions of Poland

Studies among women with abnormal cytology indicate a high prevalence of hrHPV across Polish regions, with HPV16 consistently dominant (Table 2). In this context, data from the Wielkopolska Region (central–western Poland) among women with cytological abnormalities (koilocytosis, LSIL, HSIL) demonstrate a clear genotype-specific stratification associated with lesion severity [30]. In koilocytosis, HPV16 was the most frequently detected genotype (10.9%), followed by HPV52, HPV53, and HPV66 (each 4.3%), and HPV31 and HPV51 (each 2.2%). In LSIL cases, HPV16 remained predominant (22.5%), with substantial contributions from HPV52 (9.9%), HPV31 (8.6%), HPV51 (7.9%), HPV66 (7.3%), and HPV53 (6.6%). A further increase in HPV16 prevalence was observed in HSIL (54.2%), accompanied by HPV31 (16.9%), HPV51 (9.0%), HPV52 (7.8%), HPV66 (5.4%), and HPV53 (4.2%). These findings indicate a progressive enrichment of HPV16 and HPV31 with increasing lesion severity, alongside a relative decrease in genotype diversity in high-grade lesions. These findings indicate a progressive enrichment of HPV16 and HPV31 with increasing lesion severity, alongside a relative decrease in genotype diversity in high-grade lesions. This trend is further supported by Bardin et al. [31] and Kędzia et al. (2010) [33], both of whom conducted their studies in central Poland (including the Warsaw region) and in the case of Kędzia et al. in western Poland. Additionally, Kędzia et al. [33] highlighted a greater contribution of HPV33 to CIN-1 changes.

In turn, Bebyn et al. [34] investigated the prevalence of hrHPV genotypes in cervical cytology samples from the Bydgoszcz region. Among 1840 women, most showed abnormal cytology, including LSIL, HSIL, ASC-US (atypical squamous cells of undetermined significance), ASC-H (atypical squamous cells (HSIL cannot be excluded)), and AGC (cervical glandular cells with atypical glandular cells). mRNA analysis using NucliSENS EasyQ HPV revealed that 36.3% (198 women) of the general population were HPV RNA positive. The most common genotypes were HPV16 (48.0%), HPV18 (12.6%), HPV31 (10.1%), HPV33 (8.6%), and HPV45 (4.5%), with 16.2% of women infected with more than one HPV type. Kiwerska et al. [35] analyzed data from the Warsaw (central Poland) and Poznań (western Poland) regions in 2019. They found a 100% hrHPV positivity rate in this group, with HPV16 (35.5%) being the most prevalent, but also identifying genotypes such as HPV42, HPV39, HPV54, and HPV53, which were less commonly reported in nationwide data. In turn, Nowakowski et al. [36] in the analysis covering southeastern Poland (Lublin and Krakow), found high hrHPV prevalence in women with high-grade CIN (96.1%), with HPV16 (62.8%) and HPV33 (7.8%) being the most common. Although not a general population study, it supports regional data indicating a high HPV16 burden in eastern Poland. Szostek et al. [32] provided a more nuanced analysis from southern Poland by separating women into cytological categories (LSIL, HSIL).

Taken together, data from different Polish regions consistently demonstrate a clear and reproducible pattern of HPV epidemiology characterized by a strong predominance of HPV16 across all grades of cervical lesions, with increasing frequency in higher-grade abnormalities. This dominance is accompanied by a gradual enrichment of HPV31 and a reduction in overall genotype diversity as lesion severity increases. At the same time, there is marked regional variability in the distribution of secondary high-risk HPV genotypes, including HPV31, HPV33, HPV45, HPV51, HPV52, HPV53, and HPV66, whose prevalence differs across studies and cytological subgroups. Additional regional heterogeneity is reflected in the occasional detection of less frequently reported genotypes such as HPV39, HPV42, and HPV54, further emphasizing that while HPV16 remains consistently dominant, the broader HPV genotype landscape shows substantial geographic and population-based variation within Poland. However, these results should be interpreted with caution, as the observed regional differences may also reflect inter-study variability in population characteristics (including age of patients), sampling variation, and differences in HPV detection methods and genotyping.

**Table 2:** Regional Distribution and Prevalence of High-Risk HPV Genotypes in a Patient Population With Cervical Abnormalities across Different Regions of Poland.

Authors (Year of Publication)	Cohort	Geographical Region	Material	Method of HPV Detection	hrHPV Prevalence
Przybylski et al. (2023) [30]	363 pts with: koilocytosis (*n* = 46) LSIL (*n* = 151) HSIL(*n* = 166) tested between 2018 and 2022	Wielkopolska Region (central– western Poland)	Cervical cytology material	HPV DNA Immunoassay with oligoprobe cocktails, detection of 37 HPV genotypes	**General percentage of hrHPV positivity:** **no data** **Patients with koilocytosis** HPV16—10.9% HPV52—4.3% HPV53—4.3% HPV66—4.3% HPV31—2.2% HPV51—2.2% **Patients with LSIL** HPV16—22.5% HPV52—9.9% HPV31—8.6% HPV51—7.9% HPV66—7.3% HPV53—6.6% **Patients with HSIL** HPV16—54.2% HPV31—16.9% HPV51—9.0% HPV52—7.8% HPV66—5.4% HPV53—4.2%
Bebyn et al. (2022) [34]	531 pts with HPV DNA positive test and LSIL, HSIL, ASC-US, ASC-H, AGC, tested between 2012–2014	Bydgoszcz region	Cervical cytology material	HPV DNA analysis AMPLICOR^®^ HPV Detection kit + mRNA analysis NucliSENS EasyQ HPV (detection of five hrHPV genotypes)	**General percentage of hrHPV positivity:** **198 (36.9%)** HPV16—47.9% HPV18—12.2% HPV31—10.2% HPV33—8.7%, HPV45—4.6%
Kiwerska et al. (2019) [35]	126 pts with CIN I CIN II tested between 2014 and 2016	Warsaw and Poznan regions	Cervical cytology material	Anyplex^TM^II HPV28 Detection system, Segene	**General percentage of hrHPV positivity:** **83 (65.9%)** HPV16—23.8% HPV31—17.4% HPV39—10.3% HPV54—10.3% HPV42—4.0%
Nowakowski et al. (2014) [36]	205 pts with HG-CIN tested between 2001 and 2008	Lublin and Krakow regions	FFPE	SPF_10_-DEIAliPA_25_-polymerase chain reaction (PCR) system	**General percentage of hrHPV positivity:** **197 (96.1%):** HPV16—62.8% HPV33—7.8% HPV31—6.6% HPV52—3.7% HPV45—2.6% HPV58—2.6%
Kędzia et al. (2010) [33]	126 pts with CIN-1, tested between: no data	central and western Poland	Cervical cytology material	INNO-LiPA HPV Genotyping	**General percentage of hrHPV positivity:** **no data** HPV16—54.0% HPV18—16.7% HPV31—10.3% HPV33—21.4% HPV45—7.9% HPV52—1.6% HPV39—0.8% HPV42—0.8% HPV55—0.8% HPV56—0.8% HPV58—0.8% HPV59—0.8% HPV62—0.8% HPV66—0.8% HPV73—0.8%
Bardin et al. (2008) [31]	35 pts with: atypical squamous cells (*n* = 10), LSIL (*n* = 22), HSIL (*n* = 3) tested in 2006	Warsaw region	Exfoliated cervical cells and cervical cancer biopsy	HPV DNA Immunoassay with oligoprobe cocktails, detection of 44 HPV genotypes	**General percentage of hrHPV positivity:** **23 (65.79%)** HPV16—25.7% HPV31—5.7% HPV33—5.7% HPV39—5.7% HPV51—5.7% HPV52—5.7% HPV58—5.7% HPV82—5.7% HPV18—2.9% HPV35—2.9% HPV45—2.9%
Szostek et al. (2008) [32]	56 pts with LSIL (*n* = 44), HSIL (*n* = 12), tested between: no data	Krakow region	Cervical cytology material	INNO-LiPA HPV Genotyping	**General percentage of hrHPV positivity:** **L-SIL population:** **43 (98.0%) hrHPV:** HPV51—30.2% HPV52—25.6% HPV18—11.6% HPV31—11.6% HPV16—9.3% HPV33—7.0% HPV58—7.0% HPV66—7.0% HPV56—4.6% HPV35—2.3% HPV39—2.3% **H-SIL population:** **11 (92.0%) hrHPV:** HPV16—63.6% HPV51—27.3% HPV18—18.2% HPV31—9.1%

Abbreviations: AGC, Cervical Grandular Cells with Atypical Glandular Cells; ASC-H, Atypical Squamous Cells (HSIL cannot be excluded); ASC-US, Atypical Squamous Cells of Undetermined Significance; CIN, Cervical Intraepithelial Neoplasia; HPV, Human Papillomavirus; hrHPV, High-Risk Human Papillomavirus; HSIL, High-Grade Squamous Intraepithelial Lesion; LSIL, Low-Grade Squamous Intraepithelial Lesion; pts, patients.

### 3.4. High Risk Human Papillomavirus Prevalence and Genotype Distribution in the Female Population with Invasive Cervical Cancer Across Different Regions of Poland

Table 3 summarizes findings from four studies that examined the prevalence and genotype distribution of hrHPV in women with invasive cervical cancer (ICC) across different regions of Poland, conducted between 1997 and 2008. While all studies consistently report a high prevalence of hrHPV among cervical cancers (ranging from 70.1% to 100%), regional differences may be observed in both detection rates and genotype distribution. HPV16 emerged as the predominant genotype in all regions, with the highest prevalence observed in the Gdańsk region (88.0%) [37] and southern Poland (81.4%) [32]. In the study from the Gdańsk region, which included 107 women diagnosed with cervical cancer, HPV DNA was detected in 70.1% of cases using PCR-based analysis targeting the E6/E7 regions. Among HPV-positive samples, HPV16 was identified as the dominant genotype, accounting for 88.0% of infections. Most patients were diagnosed with stage IB (43%) or IIA (19.6%) cervical cancer, and no statistically significant differences in HPV positivity were observed between clinical stages. In the study from Southern Poland [32], 27 women with diagnosed ICC were included, which allowed comparison of HPV genotype distribution across cervical cancer, precancerous lesions (Table 2), and cytologically normal controls (Table 1). In the ICC subgroup, HPV DNA was detected more frequently than in the control group, confirming the strong association between HPV infection and cervical carcinogenesis. The analysis also showed that single-type infections were more common in ICC, whereas multiple HPV infections predominated in women with LSIL and in cytologically normal samples. In addition, infection with HPV16 was strongly associated with a significantly increased risk of HSIL and ICC compared with LSIL. At the same time, the presence of other high-risk genotypes (such as HPV51 and HPV52) in ICC indicates that the genotype distribution is not completely homogeneous.

The prevalence of HPV18, the second most common oncogenic type, varied geographically—from 5.7% in the Warsaw region [32] to 11.1% in southern Poland [32]. Some regional differences may reflect methodological variability. However, they may also suggest genuine geographic heterogeneity in circulating HPV genotypes. In the Warsaw region, Bardin et al. identified a relatively diverse hrHPV profile, detecting HPV16, HPV18, HPV31, HPV45, HPV52, and HPV56 [31]. In contrast, the Lublin and Krakow regions [36] showed a narrower spectrum, with HPV16, HPV18, HPV33, and HPV45 being most frequently detected. Similarly, in the Gdańsk cohort [37], only five hrHPV types were identified, and a significantly lower overall hrHPV positivity rate (70.1%) was reported—potentially due to earlier testing technologies and limited genotype panels. This variability highlights the importance of comprehensive HPV surveillance across diverse geographical regions to better understand the regional burden of different hrHPV types.

Taken together, these findings underscore the predominance of HPV16 and HPV18 in cervical cancer across Poland but also point to regional heterogeneity in less common hrHPV genotypes. Such geographic differences are important to consider when designing regionally tailored prevention strategies, including screening and vaccination programs, especially for non-16/18 HPV types that may gain prominence as vaccination alters HPV type distribution.

**Table 3:** Regional Distribution and Prevalence of High-Risk HPV Genotypes in the Patient Population with invasive cervical cancer across different regions of Poland.

Authors (Year of Publication)	Cohort	Geographical Region	Material	Method of HPV Detection	hrHPV Prevalence
Nowakowski et al. (2014) [36]	193 pts with ICC tested between 2001–2008	Lublin and Krakow regions	FFPE blocks	SPF_10_-DEIAliPA_25_-polymerase chain reaction (PCR) system	**General percentage of hrHPV positivity:** **176 (91.2%)** HPV16—72.1% HPV18—10.8% HPV33—5.7% HPV45—3.4% HPV31—1.7%
Bardin et al. (2008) [31]	88 pts with ICC tested in 2006	Warsaw region	Exfoliated cervical cells and cervical cancer biopsy	HPV DNA Immunoassay with oligoprobe cocktails, detection of 44 HPV genotypes	**General percentage of hrHPV positivity:** **87 (98.8%):** HPV16—74.7% HPV18—5.7% HPV45—5.7% HPV31—3.4% HPV52—3.4% HPV56—3.4%
Szostek et al. (2008) [32]	27 pts with ICC tested between: no data	Southern Poland	Cervical cytology material	INNO-LiPA HPV Genotyping	**General percentage of hrHPV positivity:** **27 (100.0%)** HPV16—81.4% HPV51—14.8% HPV18—11.1% HPV66—11.1% HPV45—2.2% HPV68—2.2%
Liss et al. (2002) [37]	107 pts with ICC tested between 1997 and 1998	Gdańsk region	Cervical cytology material	DNA PCR	**General percentage of hrHPV positivity:** **75 (70.1%)** HPV16—88.0% HPV31—4.0% HPV33—4.0% HPV45—1.3% HPV52—1.3%

Abbreviations: FFPE, Formalin Fixed Paraffin Embedded; HPV, Human Papillomavirus; hrHPV, High-Risk Human Papillomavirus; pts, patients.

## 4. Interpretation of Findings

This systematic review confirms the predominance of HPV16 in Poland while bringing together and directly comparing regional datasets from across the country that have not previously been analyzed collectively in a single framework. This integrated approach is particularly important in light of existing evidence, including the nationwide study by Glińska et al. [29], which analyzed samples from 556 sites across all 16 voivodeships and provides the most comprehensive overview of hrHPV genotype circulation in Poland, but does not allow for type of changes in the cervix and region-level interpretation.

Across studies conducted in different geographical regions of Poland, HPV16 was the most frequently detected genotype in nearly all analyzed groups, including women without cytological abnormalities, women with cervical abnormalities, and patients with cervical cancer. In one study from southern Poland, concerning women without cervical abnormalities, HPV51 was most common [32] (see Table 1). Across all analyzed groups of women, variability in the prevalence of other hrHPV genotypes highlights their heterogeneous distribution. However, the question of whether these differences reflect the geographical variation in the prevalence of individual hrHPV genotypes in Poland remains open, because the results of individual studies may also be influenced by other factors, such as population composition and study design.

Population-based screening studies reported lower hrHPV prevalence (39.0% in the nationwide cohort [29] than studies focusing on women with abnormal cytology (range: 36.9% [34]—98.0% [32] or with ICC, where hrHPV prevalence ranged from over 70.1% [37] to 100% [32]. Genotype distribution also varied with lesion severity: HPV51 and HPV52 predominated in LSIL, whereas HPV16 was dominant in HSIL and high-grade CIN, consistent with its higher oncogenic potential [32,36]. In low-risk populations, such as women with normal cytology, hrHPV prevalence was substantially lower, and HPV16 was rarely detected compared with patients presenting with cervical pathological changes, including those with ICC. Another important population key factor is the age of the woman. Epidemiological data consistently show a bimodal age-specific pattern, with the highest HPV prevalence in young women shortly after sexual debut, followed by a decline in middle age and, in some populations, a secondary smaller increase in older women [6,38,39,40]. This pattern is mainly driven by differences in sexual exposure, cumulative risk, and immune system efficiency across the lifespan. Younger women more frequently present with transient HPV infections and a broader spectrum of high-risk genotypes, whereas persistent infections—more strongly associated with neoplastic progression—become relatively more common with increasing age. In older age groups, infections are less frequent overall but are more often dominated by high-risk types, particularly HPV16, which shows strong persistence and oncogenic potential across all age categories. These age-related dynamics also influence genotype distribution in cervical lesions, with some studies indicating greater genotype diversity in younger women and increasing HPV16 predominance in older patients [40,41,42,43]. Finally, the impact of HPV vaccination programs on genotype prevalence and age-specific distribution patterns should be briefly considered, as these interventions are known to significantly modify HPV epidemiology, particularly in younger populations. Population-based studies from the United States have demonstrated substantial reductions in the prevalence of vaccine-targeted HPV genotypes, especially HPV16 and HPV18, following vaccine introduction, alongside a marked decline in cervical precancerous lesions in young women [44,45,46]. These findings are supported by large systematic reviews and meta-analyses confirming strong population-level effects and herd immunity associated with HPV vaccination programs [11,47]. In addition, evidence suggests that reductions in vaccine-type HPV infections may be accompanied by relative shifts in genotype distribution, with an increased proportion of non-16/18 high-risk HPV types observed in vaccinated individuals and certain population subgroups [40]. Overall, HPV vaccination has a profound effect on both genotype prevalence and age-specific patterns of HPV infection.

Another major factor affecting inter-study comparability is the diversity in HPV detection techniques. Across the studies, there is significant heterogeneity in diagnostic platforms. The studies employed a variety of diagnostic platforms, ranging from PCR-based assays and hybrid capture techniques to mRNA analyses and multiplex genotyping systems (e.g., Anyplex II, INNO-LiPA, Linear Array, Anyplex^TM^ HPV28). These methodological differences affect both sensitivity and the spectrum of detectable HPV genotypes. For instance, broad-spectrum, type-specific PCR assays, as used by Glińska et al. [29] and Kiwerska et al. [35], identified a more diverse high-risk HPV (hrHPV) profile, including less common genotypes such as HPV53, HPV42, and HPV68. Conversely, mRNA-based assays (e.g., NucliSENS EasyQ used by Bebyn et al. [34]) target a limited number of genotypes but detect active viral transcription, potentially underestimating overall prevalence. Earlier studies [31,36] relying on DNA hybridization or older PCR methods generally exhibited lower sensitivity and narrower genotype coverage. These methodological disparities likely contribute to variations in reported prevalence and bias genotype frequency estimates toward types detectable by the respective assays. Importantly, rarer genotypes may be underrepresented in older or less comprehensive studies. To improve comparability and reliability of HPV surveillance data globally, standardization of detection methods and assay platforms is critically needed in future research. Standardization of HPV testing platforms in future surveillance studies is essential to ensure consistency, reproducibility, and data comparability across regions and over time.

Based on the current body of evidence and existing knowledge gaps, several critical priorities have been identified to advance our understanding and control of HPV-related disease in Poland. First, there is a pressing need for longitudinal, multicenter cohort studies that can track changes in HPV prevalence over time. Such studies are particularly important in the context of widespread vaccination programs, as they will help monitor vaccine impact and detect any shifts in genotype distribution, including the potential rise in non-vaccine HPV types in the post-vaccination era. Second, regional surveillance efforts must be reinforced to reflect the known geographic variability in the distribution of high-risk HPV genotypes. Strengthening these programs will facilitate tailored screening strategies and vaccination policies that are responsive to local epidemiological patterns. Third, the adoption of standardized genotyping protocols across national cervical cancer screening programs is essential. This standardization will ensure consistent and comparable data collection, improve data quality, and promote better integration with electronic health records and public health databases. Additionally, broader inclusion of diverse population groups in surveillance and research is crucial. Special attention should be given to underserved or high-risk populations, such as women living in rural areas, older women, and immunocompromised individuals, to ensure equitable access to prevention and care services. Finally, linking molecular HPV findings with clinical outcomes—including viral persistence, clearance rates, and progression of precancerous lesions—will enhance our understanding of the carcinogenic potential of less common high-risk HPV genotypes beyond types 16 and 18. This knowledge is vital for refining risk stratification and optimizing patient management in cervical cancer prevention programs.

The observed potential regional variability in hrHPV genotype distribution in our review may influence the performance and interpretation of HPV-based screening programs, especially in the context of the recently introduced primary HPV testing. Furthermore, these data may be relevant for evaluating the potential real-world effectiveness of current vaccination strategies and for considering whether future public health policies should incorporate more regionally informed surveillance or targeted prevention approaches. It should be understood that the introduction in Poland of a primary cervical cancer screening model based on the detection of DNA from oncogenic HPV types for women between 25 and 64 years, implemented within a program funded by the National Health Fund, represents an important step toward modern, evidence-based secondary prevention. This strategy is consistent with current international recommendations and reflects the well-documented higher sensitivity of HPV testing compared with conventional cytology. At the same time, analysis of the epidemiological data presented in this study suggests that the performance of this screening model may be partially influenced by local biological factors and regional heterogeneity in circulating hrHPV genotypes. Specifically, differences in the distribution of high-risk HPV types across populations may affect the sensitivity of HPV-based screening, as most screening algorithms are primarily optimized for detecting the most oncogenic genotypes, particularly HPV16 and HPV18. In regions where other hrHPV types (e.g., HPV31, HPV52, HPV53, or HPV66) contribute more substantially to the overall infection burden, the proportion of lesions associated with non-16/18 genotypes may be relatively higher, which can influence detection patterns and risk stratification. In addition, host-related biological factors may further modulate screening performance by influencing susceptibility to HPV infection, viral persistence, and the likelihood of progression to clinically significant cervical lesions. These factors include individual immune response variability, age-related differences in immune surveillance, hormonal status, and the composition of the cervical microbiome, all of which may affect whether HPV infections are transient or persistent. Consequently, the same screening algorithm may exhibit different performance characteristics across populations, not only because of viral genotype distribution but also because of underlying host-dependent differences in infection dynamics and disease progression.

Although HPV16 remains the dominant genotype across all regions of Poland and across all disease stages, the distribution of other hrHPV types shows meaningful geographic variation. In southern Poland, HPV51 has been reported relatively frequently, including in LSIL and HSIL lesions [32], whereas studies from central regions more often identified HPV56 and HPV45 [31]. More recent nationwide studies additionally indicate an increasing prevalence of HPV31 and HPV66, highlighting the dynamic nature of hrHPV epidemiology over time [29]. In the context of an HPV-based primary screening algorithm with cytology performed only in HPV-positive women, this regional diversity of hrHPV genotypes underscores the importance of adequate test sensitivity, broad genotype coverage, and the quality of cytological triage. These observations do not challenge the rationale for the current screening model but rather suggest potential value in continuing to optimize it based on national and regional epidemiological data, which may further enhance the program’s effectiveness and clinical safety. On the other hand, since 2023, Poland has implemented a national HPV immunization program to procure and distribute HPV vaccines for children aged 12 to 13 years [48]. Updated recommendations introduced in 2024 expanded program eligibility to all children between 9 and 14 years [49]. Data from 2025 revealed that only a small number of children (8.67%) in the relevant age groups received the HPV vaccine, indicating persistently low vaccination coverage within the pediatric population [50]. This low coverage suggests that, despite evidence from countries such as the United States demonstrating clear population-level benefits of high-coverage vaccination programs (substantial reductions in the prevalence of vaccine-targeted HPV genotypes, particularly HPV16 and HPV18 in young women) [51], similar epidemiological impact in Poland will likely be delayed until vaccination uptake increases substantially and sustained high coverage is achieved across successive birth cohorts. Moreover, the occurrence of HPV-related disease “despite vaccination” is expected due to other factors:

1. Latency and pre-existing infection: HPV vaccination does not treat existing infections. Therefore, HPV-related disease (e.g., cervical dysplasia or cancer) may still develop in individuals infected prior to vaccination or before achieving full immunological protection.
2. Partial type coverage: Current vaccines primarily target high-risk HPV types (notably HPV 16 and 18, and in the nonavalent formulation, additional types), but do not cover all oncogenic HPV genotypes.
3. Time lag effect: The preventive impact on cervical cancer incidence is delayed by years to decades due to the long natural history of HPV-induced carcinogenesis.

Therefore, it should be understood that the continued presence of HPV-related disease in Poland is consistent with expected epidemiological dynamics during early and intermediate phases of vaccine program implementation, rather than evidence of vaccine failure.

## 5. Conclusions

Although HPV16 remains the predominant oncogenic type across all regions and population groups in Poland, significant inter-study variation in genotype distribution exists, probably influenced by regional factors, study design, and diagnostic methodology. These findings underscore the importance of standardized, geographically comprehensive surveillance and the need for future studies that integrate epidemiological, molecular, and clinical data to inform effective and adaptive public health interventions, particularly in the era of HPV vaccination.

## Data Availability

The data are available from the corresponding author upon reasonable request.
